# What Have Games Got to Do with Me?

**DOI:** 10.1016/j.patter.2020.100002

**Published:** 2020-03-20

**Authors:** Valerie McCutcheon, George Bray

**Affiliations:** 1 Information Services, University of Glasgow, Glasgow, Scotland, G12 8QE, UK; 2 Library, Robert Gordon University, Aberdeen, Scotland, AB10 7GJ, UK

## Abstract

Is the proliferation of work-based games just a distraction, or can they actually help us to acquire work-specific knowledge? This Opinion explains why we can see the benefits of such games, despite initial skepticism. Players learn from listening to and observing others, and some people even enjoy the games.

## Main Text

### Introduction

Researchers are often busy people who are under significant time pressure and managing hefty workloads. It can therefore be a challenge for them to find the time to engage with the myriad requirements and opportunities that are relevant to what they do. Over recent years, games that are intended to foster engagement with good research practice, skills, and knowledge have created a bit of a buzz among my peers. The question is whether games can help to facilitate the efficient sharing of information and provision of services between research support staff and researchers, easing the dissemination of best practices, skills, and knowledge throughout the community. An additional question is whether such games can also serve as useful tools when used only within the research support community, to help identify gaps in the knowledge and services of research support staff.

As a self-confessed “venatophobian,” I’ll admit that I was skeptical. However, if we were able to demonstrate that games-based interactions could provide a successful channel of communication, perhaps I could swallow my unease and get on board. So, when my friend George Bray from Robert Gordon University mentioned one of these games at an Open Access Scotland meeting, I heard myself suggesting that we host a games day to explore how games might help us in supporting researchers.

When preparing to run the event, we did some research into what games might be relevant, selecting a few that were specifically related to open research practices. In order to be considered viable for the event that we had in mind, the proposed games or games-based sessions had to:

• be a game or a games-based session—some things advertise themselves as “games” but don’t include any game mechanics and are just interactive tools for research support (a valid approach, but not in scope of what we had in mind for the event);
• be relevant to educating players on best practices, skills, or knowledge for some aspect of open research;
• be primarily intended for helping with researcher education, rather than support staff education; and
• have someone (preferably the game’s designer) be willing and available to showcase and facilitate the game at the event.

Our reasoning for choosing open research as the main focus was that it is a central aspect of both our roles in our respective universities—for example, I lead a team that delivers open access and research data management support and am also heavily involved in the broader research support community through such things as the Open Access Scotland group and by being the Open Access Special Interest Group champion for the Association of Research Managers and Administrators (ARMA).

We eventually settled on a single-day event, consisting of three 1-h slots for gameplay as well as a group discussion to finish the day. We planned to try seven different games:

• Curate! The Digital Curator Game (Output of a European Commission project), http://schreibman.eu/digcurv/curate-game/
• The Game of Open Access (University of Huddersfield), https://hud.libguides.com/openaccess/GameOfOpenAccess
• The Impact Game (Cranfield University), https://www.ivorygraphics.co.uk/shop/games/3075/the_impact_game
• LEGO: Metadata for Reproducibility (University of Glasgow), https://doi.org/10.36399/gla.pubs.196477
• The Open Access Escape Room (University of Essex), https://figshare.com/projects/Open_Access_Escape_Room/56915
• The Publishing Trap (UK Copyright Literacy), https://copyrightliteracy.org/resources/the-publishing-trap/the-publishing-trap-resources/
• A workshop on how to make virtual games

These were split up across the available timeslots and among the six tables (each hosting a maximum of six attendees).

After advertising the event through Eventbrite and via several mailing lists, we were surprised by the high level of interest and discussion that was provoked by our advert. All tickets were quickly reserved—in fact, we had to increase the number of tickets to equal the venue’s maximum capacity (36 attendees), as we originally underestimated how many people would want to attend. Participants were mainly research support staff or lecturers and came from across the UK. We therefore had a packed room when, in September 2019, we hosted our first workshop to explore our key question: how useful could games really be as tools for communicating open research best practices, skills, and knowledge? Here are my thoughts on the day.

### Overview

Most sessions ran twice over the course of the day, with multiple different games or workshops running simultaneously at different tables in each hour-long slot of gameplay. This meant that all attendees were able to try at least two or three different sessions and that facilitators were running sessions for tables of between four and six attendees. Feedback was solicited in a discussion session at the end of the workshop, based on a questionnaire that was made available to attendees throughout the day. Respondents were asked to give brief reviews of the sessions that they had attended, focusing on positives and constructive criticism. Additionally, respondents fed back on broader questions, like the following:

What are the advantages of using games-based research support?
What makes games fun for you?
What are the potential issues that should be considered when using games as part of a research support service?
What do we want to see more games about?

Most attendees seemed to agree that games are a good way to engage with researchers on dry topics and to foster conversations on challenging aspects of the research landscape. People felt that the informal nature of games was likely to be especially helpful with postgraduates and early-career researchers. The innate interactivity of games was the main reason why people found them to be useful ways of increasing engagement and information retention.

It was noted that some of the games needed additional resource from support staff, in order to customize them to fit local policies. Moreover, adequate preparation for a game session was identified as being key to its success. A multi-disciplinary group of players might foster connections and share different views. It was also recognized that running a session with players from only a single discipline would be useful in some cases, to help keep the topic focused.

We also recognized that games are not enjoyed by everyone. It was therefore suggested that facilitators need to be prepared to handle those session attendees who prefer to spectate, finding other ways to involve them in the discussions arising from gameplay.

A summary of the games chosen and the feedback are available here: http://eprints.gla.ac.uk/197588/^1^

### Observations on Specific Sessions

#### Virtual Games Group

For my first session, I joined the virtual games group because it felt like the most comfortable option for someone who is as disinclined to engage with “traditional” games as I am. We were building our own computer games, and I certainly felt less inclined to run from the room after I had changed the Dragon Realm example into something more meaningful for me.

The reference for this session was the book *Invent Your Own Computer Games with Python* by Al Sweigart, which is available open access online.^2^ We used Python Anywhere, a cloud version, to get started in writing some code, dredging up “Hello World” from the dusty recesses of what I remembered about learning how to code and giving it a bit of a makeover, so that it became “Hello Games Day!” For the rest of the session, the example that we worked on was code that asked a question about whether to enter a cave. Depending on the choice taken, you might meet a fiery dragon. Once the coding technique was learned with simple examples, we amended the code into a real question that might be used as part of an online training tool, such as “Do you need to get ethics approval to interview members of the public?” There are templates available, and there is a lot of code shared in GitHub.

I surprised myself because it was barely time for the first coffee break and I could already see some potential value. We were doing something fun that could also have a practical use for research support—a view that was echoed by other attendees throughout the day. I could see that we could build some fun ways for service users to learn about support options while also reducing their administrative burden.

I was asked about room temperature, and as I headed off to find a control panel, I was suddenly targeted by a few people who thought I might be taking part in the Open Access Escape Room game. Don’t follow *me* for clues, folks!

#### Digital Preservation

Having exhausted my courage, I decided to stay away from active participation and instead moved on to observe the Curate! game, which you can find online here: http://schreibman.eu/digcurv/curate-game/. It’s a bit like Monopoly for digital preservation, with instructions on squares that direct player actions. Some of the prompts included taking a card (either “danger,” “caution,” or “DigCurV”), missing a turn, or going back a space. Players worked together to answer questions and to gather best practices.

The aim was to discuss good practice in planning and running digital-preservation activities from a strategic viewpoint. This encouraged some useful discussions about the decisions required if the players were to be given a budget for digitization. Which material would they select and why? Would it be shared and, if so, with what license?

I think the game has the potential for general application, regardless of your project topic. It showcases the importance of developing a business case, planning carefully, and communicating clearly in order to achieve your goals. After listening in, I noted that it seemed to be a wholeheartedly supportive discussion, with no one appearing uncomfortable in using the game to facilitate the more serious conversation. Perhaps even I could be tempted to have a go?

#### The Game of Open Access

In the final session, I observed the Game of Open Access. The game is intended to help research support staff engage with authors and to prompt discussion on issues around open access. The version used for the session had been based around local policy and procedures at the University of Huddersfield; however, it is possible to download the materials and customize them for your own institution here: https://hud.libguides.com/openaccess/GameOfOpenAccess.

By throwing dice and moving tokens around the board, the game demonstrated scenarios that might occur when publishing an article—you might find your token landing on a square that featured such a scenario or one that required you to draw a card that posed a question relating to a commonly encountered issue. In reading out these scenarios and thinking about answers to questions, we also wandered, usefully, into broader discussions on associated topics, such as data-management support and costs of storage. This was great and demonstrated how the game made an excellent conversation starter, leading to deeper discussions that help to support authors and the sharing of best practices between research support practitioners.

Do I want to play it? No. However, do I think it would be a useful tool for us, and other teams, to use at our own institutions? Yes, and I would be happy to facilitate such sessions.

### Conclusion

Sadly—and I was sad—I did not have time to try all the games on offer during the day. However, I feel that my curiosity was rewarded, and my perspective has changed; I can now see that the context of a game sometimes provides people with a platform for discussion, to which they may not otherwise have access.

There were many games that we could have included but for which we did not have capacity. For example, one attendee suggested adopting the Cards Against Humanity game, customizing the cards to facilitate research-related conversation with a strong element of humor. In my opinion, the most successful games are those that are both intuitive to play and easily customizable. We were glad to have helped promote the games that we did, providing a range of options that our research support audience were keen to try. We are also glad to have so many other games that we did not use in this first event, as it means that we would have plenty of content for a future iteration of the Games Day.

Looking ahead, I have been in touch with representatives of similar events; I hope that we can collaborate in future, sharing event outputs as well as best practices for hosting such events. We have also discussed the possibility of having some sort of online resource, not tied to any organization or game, which would host links to the many games and support materials that are out there. For example, one peer has set up a Wakelet (“Research and publishing games,” https://wakelet.com/wake/db1a9d90-a44c-4e08-8007-24d622bf9aa1), which I look forward to utilizing as a go-to place for storing and finding details of games.^3^

The workshop was such a success that I am looking forward to our next games day. I might even find myself joining in more, as we create play options specifically for our audience.

If you want a reading recommendation, I am currently enjoying the newly published *Graduate Skills and Game-Based Learning* by Matt Barr,^4^ a colleague of mine from previous projects. The book focuses on online games, but there are many observations that resonate with our recent games experience. I’m hoping it will provide inspiration for our next session.

I also watched my niece play *Fortnite*, and while I had no desire to play, I was struck by how even a non-educational game can give a young child an immediate sense of strategic planning and the need to support all team members. (I am not convinced that *Just Dance* had the same impression on me, but hey, it was fun and not too intimidating.)

Games, I admire you.
